# European Correspondence

**Published:** 1878-09

**Authors:** R. J. Nunn

**Affiliations:** Paris, France


### EUROPEAN CORRESPONDENCE.

Paris, France, July 22, 1878.

Surgery is a branch of the art curative in which national peculiarities may be very distinctly seen; and while, according to first-class surgeons of each country, the same amount of operative skill and an equal amount of scientific acquirements are necessary, the operative manner of the surgeons of two races appears to be absolutely distinct and different, and becomes almost national in its character.

I was led into this vein of thought by seeing the difference between the operating rooms here and in London. I very well remember being present at some operations performed by one of England's most distinguished surgeons. Around the operating table whereon lay the patient were grouped the surgeons, two assistants, the anæsthetizer, and a nurse to pass sponges, and these never left the positions in which they were placed, nor did one at any time appear to undertake the duties of the other. Two other nurses were in the room to wash and carry sponges, etc, and a half dozen privileged spectators, to whom were assigned more elevated positions, completed the number of witnesses. During the operation not an audible word was spoken. A whisper may have passed now and then between the operator and his assistants, but there was no talking. Instruments, etc., appeared to have been thoughtfully placed just where they would be most convenient, and everything seemed to be at hand just when wanted, without the necessity of asking for it. Silence was, as I have observed, strictly maintained—I might say enforced, for one of the visitors, venturing to indulge in a whispered conversation with his neighbor, it became audible to the surgeon, who quickly called him to order.

This is one extreme. I do not wish to convey the idea that such strictness is always observed in England—far from it; but there certainly does appear to be lines distinctly drawn, either by rule or conventionality, which the parties do not overstep.

Now let me describe the opposite extreme. The operator is a deservedly distinguished Parisian surgeon. His assistants, to the number of seven, with two nurses, await him in his operating theater, which is well filled with spectators. After a lucid explanation of the case and the methods to be adopted, the operation commences. Chloroform has been administered, and the breathing becoming a little suspicious, the tongue has been seized with a forceps, and now, as the operation proceeds, it becomes evident that the anæsthetizer is a student, and more interested in the progress of the case than in the particular department to which he has been assigned; and so the patient's head lolls to one side, partially suspended as it were by the tongue, dragged out at one corner of the closed mouth. This does no harm, it is true, but it makes an unpleasant impression on the spectators. Then, as the operation proceeds, it will be observed that the instruments have been placed inconveniently at a considerable distance from the operator, so that he has to call for what he wants perhaps twice or three times; then, when he is done with an instrument for the present, it is not replaced in its proper place, so that the next time it is wanted it cannot be found. Next, the sponges appear to be wrong; they are not the right size—either too large or too small; they are too wet, they are not at hand when wanted, or the assistant does not use them at the proper time. To remedy this, all the assistants begin to pass instruments and sponges, and then the spectators become interested, and some of them invade the arena. I have counted twenty interfering with the assistants and obstructing the view of those who remain in their places. All this time, notwithstanding all this hubbub and fuss, the operation is progressing grandly, the operator talking incessantly—now calling two or three times for an instrument or a sponge, now grumbling at the slowness or clumsiness of his assistants, and now explaining some difficult point he is attacking; and so the surgeon proceeds, performing his part as quickly and as dexterously as it can be done by any man in the world, and obtaining as fine results.

This, too, is an extreme case. The picture is not extravagant nor overdrawn, but it is on the other end of the line from the description of the English operating-room I have just given. Between these two there exists, of course, every possible variety, and they are expressive of a national character which is to be seen every where and in every act, and which, as I have shown, is manifested even in the operating-room of the surgeon.

The meagre accommodations offered to students cannot fail to strike an observer. The lecture-rooms of the Eeale de Medicine are a disgrace to the nation. Seats without backs, without any convenience for taking notes, no means for access except by walking on the seats, which, as an invariable result, are filthy in the extreme; and here the students sit, many of them disrespectfully keeping their hats on during the lecture, simply, I suppose, because the authorities have not provided any more convenient place to put them. In this respect Paris, which spends thousands upon thousands of franks on an illumination, is far behind the civilization of the age.

M. Bouchut has been delivering, at the "Hospital des Eufauts," a series of most interesting and instructive lectures on cerebroscopy; in other words, on the diagnosis of diseases, or conditions, of the brain, by the inspection of the eye. The theory that conditions of the brain and appearances of the eye run parallel the one with the other, the eye taking on appearances indicative of unseen changes going on in the brain; thus, whatever obstructs the flow of blood in the veins in the brain is followed by venous congestion of the retina; and so with arterial congestion, the obstruction or arrest of arterial flow in the brain is followed by a similar condition of the retina, and thus death can be infallibly distinguished from trance. Tubercle in the brain is invariably accompanied by tubercle of the retina, and so on. Many of the investigations are quite modern, and his lectures are profusely illustrated with specimens shown by the oxy-hydorgen light.

In the wards, Mr. Bouchut took much pains to show me some very interesting cases: one of purpura hemorrhagica, treated by direct transfusion. In this case the number of red globules had descended as low as 577,875, and the white had once reached a minimum of 7,613 in the M. M. cube; the normal proportion being 5,000,000 red and 6,000 white. The case is progressing favorably, and the very accurate notes which are being kept cannot fail to be of great interest, showing some curious variations in the relative proportions of red and white globlules, which appeared to oscillate independently of the actual number of either present. But probably it will be more satisfactory to give the actual numbers given to me as the results of nine examinations, and attach the proportion to each:

| | ONE CUBIC MILLIMETRE CONTAINED. | | BEING A PROPORTION OF | | |
|---|---|---|---|---|---|
| | White Globules. | Red Globules. | White Globules | to | Red Globules. |
| 1 | 8.500 | 929.625 | 1 | to | 109 |
| 2 | 7.613 | 1.407.100 | 1 | to | 184 |
| 3 | 17.901 | 2.010.000 | 1 | to | 112 |
| 4 | 9.829 | 1.482.375 | 1 | to | 149 |
| 5 | 8.200 | .854.250 | 1 | to | 104 |
| 6 | 25.115 | 678.375 | 1 | to | 27 |
| 7 | 21.775 | 778.875 | 1 | to | 35 |
| 8 | 10.887 | 978.000 | 1 | to | 89 |
| 9 | 8.375 | 577.875 | 1 | to | 69 |

According to M. M. Duval and Lereboullet, the average proportion in health of white to red globules is 1 to 350, "but" "the number of white globules tend to diminish under the influence of abstinence or advanced age (1 to 1000) it is more considerable, on the contrary after a meal, after a purgative, during pregnancy, after hemorrhages and *in children.*"

Here were also cases of albuminuria progressing towards recovery under a treatment by Fuschine, the maximum dose being 50 m grams and not the least interesting cases were different cases of chorea treated successfully, some with arsenic and others with tincture of gelsemium sempervirens.

R. J. NUNN.

